# Death of Lisfranc

**Published:** 1847-06

**Authors:** 


7. *Death of the great French Surgeon, Lisfranc.*—To the Editor: Dear Sir—I have just received a letter from my nephew Dr. F. Willis Fisher, who is now in Paris, from which I make the following extract. Thinking that you probably might be pleased to be the first medical editor who should announce the death of Lisfranc to our public, I take the liberty of sending to you the extract. You will, of course, do what you please with it.

In haste, yours truly,
*Boston, June* 7, 1847. J. D. Fisher.

"One of the most noted of the French Surgeons has paid his tribute to nature. Lisfranc is dead! After four weeks of suffering from pseudo-membranous croup (*angine couenneuse*) he fell a victim to that relentless malady. He requested that tracheotomy might be performed for his relief; but that operation was objected to, as false membranes were already formed in the pharynx, œsophagus, larynx, bronchi,

&c. He was aware of his situation, and said that he would not shrink before death *if his work on operative medicine were completed!* This remark of the dying Lisfranc is characteristic, not of Lisfranc alone, but of the French savans generally. It has often amused me to notice how jealous the French professors are of their scientific brethren, and how anxious they are to obtain professional notoriety. Lisfranc had become an old man, and he had all the feelings of youthful ambition. He had served as surgeon in the armies of Napoleon, and had been honorably noticed by that great man; and yet, on the day of his death, his craving for increased honor and glory seemed to be as imperious as ever. Lisfranc seems to have been a greater favorite with his countrymen than with foreign physicians and students. As a teacher he was much followed, and as a surgeon he deservedly held a high rank. He was a man of strong and violent prejudices—and never failed in exhibiting them in his lectures, whenever he spoke of those whom he considered as rivals. Dupuytren and Velpeau were the peculiar recipients of his censures—and he oftentimes spoke of these great men with a severity and an eloquence which must have characterized the age of the French revolution. All those who were familiar with the social character of Lisfranc, say that he was a warm hearted man—that he was ever a friend of the poor, and that his services and his purse were ever at the call of suffering humanity."—*Bost. Med. and Surg. Jour.*

